# Radiofrequency Improves Facial Fine Lines by Thermal Effect: Damage or Just Stimulation?

**DOI:** 10.1111/jocd.16600

**Published:** 2024-09-26

**Authors:** Yubing Bai, Wei Ni, Yiqiu Zhang, Zhixuan Jiang, Shengzhe Zhou, Min Yao

**Affiliations:** ^1^ Department of Plastic and Reconstructive Surgery Shanghai Ninth People's Hospital, Shanghai Jiao Tong University School of Medicine Shanghai China; ^2^ MOST Group, Wuhan National Laboratory of Optoelectronics, Britton Chance Center for Biomedical Photonics Wuhan China

**Keywords:** collagen, radiofrequency, rejuvenation, skin tightening, wrinkles

## Abstract

**Background:**

Radiofrequency (RF) has been widely used for rejuvenation treatments, but without knowledge of the effective temperature of the target tissues or guidance on treatment parameters, it often leads to adverse reactions and pain. The aim of this study was to demonstrate that thermal stimulation can produce facial rejuvenation effects and to determine the optimal bipolar RF treatment parameters for treating facial fine lines.

**Methods:**

A bipolar RF device combined with CORE Technology was used in this study. Ex vivo studies were conducted on both miniature swine and human skin, utilizing thermographic thermometry and histological analysis. In vivo swine studies were conducted using histological analysis and electron microscopy. A clinical trial was conducted, and the results were evaluated using the Alexiades Comprehensive Grading Scale (ACGS) and Global Aesthetic Improvement Scale (GAIS) scales.

**Results:**

The bipolar RF technology can produce a significant effect by thermally stimulating collagen within just 2 weeks without causing thermal damage. A clinical trial involving 46 patients showed a noticeable rejuvenating effect of the bipolar RF device, especially on fine wrinkles.

**Conclusion:**

This study confirmed that thermal stimulation, rather than thermal damage, is sufficient to achieve rejuvenation effects. The study also found a range of bipolar RF treatment parameters that are both safe and effective for facial fine lines.

## 1 Introduction

Rejuvenation is becoming an increasingly common pursuit among cosmetic patients, especially for younger patients with specific superficial wrinkles. However, the current available rejuvenation methods, such as surgery, soft tissue fillers, and botox injections, are primarily designed for patients with more severe facial aging [1]. On the other hand, laser and energy‐based technologies are in line with the trend of minimally invasive treatments [2], making them a popular option for individuals experiencing mild facial laxity looking to rejuvenate their appearance. Among them, radiofrequency (RF) is the most widely utilized for skin tightening.

A decade ago, it was discovered that RF treatment achieves facial rejuvenation by inducing thermal damage to stimulate changes in collagen conformation and promote neocollagenesis in deep layers of the skin and subcutaneous tissue, depending on the level of energy used and the impedance of biological tissues [3, 4, 5]. However, as research advances, it has been confirmed that tissue coagulation and necrosis can occur if the temperature reaches 58°C in vivo [6]. When the epidermal temperature reaches 47°C, it can cause early epidermal damage, which may lead to burns or scarring. Temperatures exceeding 70°C can even result in nerve ablation [5]. To date, we are still unsure about the temperature of the target tissues that should be controlled for RF rejuvenation treatment. Recently, through in vitro experiments, it has been found that when heat penetrates the dermis, raising the temperature to 43°C–45°C for 10–15 min promotes the proliferation of human dermal fibroblasts and the production of extracellular matrix (ECM) without causing injury to the epidermis [4, 5, 7]. Currently, several researchers recommend longer exposure time of 3–5 min with lower temperature of 43°C to prevent adverse effects such as skin burning [8]. However, there is still no study confirming that clinical effects can be achieved without any thermal damage. Therefore, identifying the appropriate temperature range and the target changes of the tissue are urgent issues that need to be addressed for RF treatment.

Our research aims to determine whether RF treatment can produce facial regeneration effects under the condition of only thermal stimulation without causing thermal damage. Additionally, we seek to identify the optimal treatment parameters to guide future RF rejuvenation treatments. This will be achieved through the use of thermal imaging, histological changes, and a clinical trial.

## 2 Materials and Methods

### 2.1 RF Device

The skin tightening (ST) applicator of the bipolar RF device (Reaction, Sinclair Co., Ltd., Israel) combines with CORE Technology, which includes three distinct RF frequency channel modes: ModeI at 0.8 MHz, ModeII at 1.7 MHz, and ModeIII at 2.45 MHz.

### 2.2 Ex Vivo

To investigate the thermal effects, the researchers treated human and swine skin tissue ex vivo by this bipolar RF. This ex vivo animal's skin was derived from the dorsal of a 6‐month‐old male Bama miniature swine weighed 25 kg. The full‐thickness skin of an ex vivo human in the study was obtained from a 35‐year‐old Asian woman under an abdominoplasty. Written consent was provided, by which the patients agreed to the use and analysis of their data.

The infrared thermometer (HIKMICRO‐H16, China) was used to monitor the skin temperature of human and pig skin tissues treated by RF (30 and 70 J/cm^3^, 0.8, 1.7, 2.45 MHz). The highest temperature reached by the epidermis or subcutaneous tissue at the corresponding time points was measured and recorded every 5 s within 20 s, and a temperature time curve was made. At the same time, changes in the heating depth of the skin were recorded by Fluke Ti480Pro portable thermal imaging camera (Fluke Thermography, MN, USA).

Following treatment, the skin tissues were fixed in 4% paraformaldehyde for 24 h, then embedded in paraffin and sectioned at a thickness of 5 μm for histological examination. The sections underwent staining with hematoxylin–eosin (HE) and Masson's trichrome using established protocols.

### 2.3 In Vivo

The procedure was performed on a mature male Bama miniature swine aged 6 months and weighed 25 kg. Experiment protocols were approved by institutional guidelines. Before the operation, researchers intramuscularly injected Xylazine Hydrochloride (8 mg/kg; Jilin Huamu Pharmaceutical Co., Ltd., Jilin, China) and sodium pentobarbital (15 mg/kg; Beijing Fangcheng Technology Co., Ltd., Beijing, China) for general anesthesia.

Before the treatment, a 2‐mm thick coupling gel was applied on treatment area. The swine was operated on the dorsal skin using bipolar RF with parameters of 60 J/cm^3^, 0.8 MHz, and 2.45 MHz, maintained for 5 s and 15 s. When heating for 20 s, the pigskin tissue was obviously burned. The researchers also used 2.45 MHz with the power of 60 J/cm^3^ for 8 passes in order to confirm the clinical effect. 8 passes means that pulses are released once per second and repeated 8 times per treatment point, with each treatment lasting approximately 7–8 s per point. The second treatment was given 2 weeks apart. After 48 h and 2 weeks of the initial treatment, as well as 2 weeks following the second treatment, pig tissues were collected and fixed using the previously mentioned procedure for histological analysis. The tissues were subjected to HE and Masson staining, and electron microscopy was also conducted.

### 2.4 Clinical Trial

The study is a single‐center single‐arm, interventional, prospective clinical study. It received approval from the Ethic Board. All patients provided informed consent before participating in the study, and they all received full‐face treatment with proper information.

### 2.5 Patients

Between May 2023 and January 2024, a sum of 46 patients were recruited. The inclusion criteria for the study included patients who were between the ages of 26 and 65 and had mild to moderate facial laxity and wrinkles. This included laxity of the eyelids, as well as wrinkles such as forehead lines and crow's feet.

The study excluded patients who were pregnant or lactating, as well as those diagnosed with severe skin laxity necessitating surgical treatment, people suffering from severe skin conditions or underlying medical conditions, as well as those who were allergic to glycerin or the coupling agent gel used for RF treatment. Patients who were unable to follow the prescribed course of treatment and those who had undergone facial chemical peel or laser treatment, or had received facial botulinum toxin injections or soft tissue fillers within the past 6 months were also excluded from the study.

### 2.6 Treatment Protocols

After inquiring about the patient's needs and assessing their facial wrinkles, a treatment plan is determined. Before treatment, the patient's face was cleaned and a 2 mm thick coupling gel was applied. Depending on the anatomical position, the operator used 2.45 MHz and repeated the treatment 6–8 times in the same area, each with a power of 50–80 J/cm^3^. The energy level was moderately adjusted based on each individual's tolerance. The objective of the treatment was to achieve a slight reddening of the skin in the treated area, while ensuring the patient felt a warm but tolerable sensation. Each operation had a duration of approximately 20 min, and a total of three sessions were administered, spaced 3 weeks apart.

### 2.7 Clinical Assessment

At each pre‐treatment and 3‐month post‐treatment follow‐up visit, patients' standardized photographs were taken and analyzed using the VISIA skin analyzer (Canfield Scientific, USA). Videos were taken by digital camera (Canon EOS 60D, Japan). When recording the video, we instructed the patients to make expressive movements such as raising eyebrows, frowning, and smiling and recording them. Facial rejuvenation was evaluated by two independent observers using the Alexiades Comprehensive Grading Scale (ACGS) (Table 1) and the Global Aesthetic Improvement Scale (GAIS) based on the photographs [9]. The ACGS is a five‐dimensional scale that assesses rejuvenation in terms of wrinkles, laxity, erythema/telangiectasia, texture, and pores. The GAIS assessment measured overall progress on a 5‐point scale, ranging from 1—“worse” to 5—“very significantly improved.” Additionally, a blinded independent observer assessed the efficacy of the treatment in various areas by analyzing changes in wrinkle length, density, and depth in baseline and post‐treatment photos. The degree of improvement was expressed as a percentage, with a higher percentage indicating more significant improvement. The treatment groups were categorized based on specific areas, such as periocular, frontal wrinkles, and frown lines, in order to determine the effectiveness of the treatment method in each region. Patients were also classified by age, with those under 35 considered young and those 35 and older classified as middle‐aged, to evaluate whether the treatment was more effective for a specific age group. Throughout the entire process, any adverse effects, such as pain, skin swelling, ecchymosis, hyperpigmentation, hypochromia, numbness, and systemic adverse events, were recorded. Besides, the level of pain that occurred during the process was assessed by Visual Analogue Scale (VAS) [10]. The satisfaction of the patients was also recorded with a total of 10 points.

**TABLE 1:** The Alexiades Comprehensive Grading Scale.

Alexiades comprehensive grading scale of wrinkles, laxity, and photodamage						
Grading scale	Parameter	Wrinkles	Laxity	Erythema/telangiectasia	Texture	Pore
0	None	None	None	None	None	None
1	Mild	Wrinkles in motion, few, superficial	Localized to NL folds	Pink E or few T, localized to single site	Subtle irregularity	Few, small, nose
1.5	Mild	Wrinkles in motion, multiple, superficial	Localized, NL and early ML folds	Pink E or several T localized to 2 sites	Mild irregularity in few areas	Several, small, nose
2	Moderate	Wrinkles at rest, few, localized, superficial	Localized, NL/ML folds, early jowels, early submental/SM	Red E or multiple T localized to 2 sites	Rough in few, localized sites	Multiple, small, nose and early medial cheeks
2.5	Moderate	Wrinkles at rest, multiple, localized, superficial	Localized, prominent NL/ML folds, jowels and SM	Red E or multiple T localized to 3 sites	Rough in several localized sites	Multiple, small, localized site (nose, cheeks)
3	Advanced	Wrinkles at rest, multiple, forehead, PO and perioral sites, superficial	Prominent NL/ML folds, jowels and SM, early neck strands	Violaceous E or many T, multiple sites	Rough in multiple, localized sites	Many, large, localized sites (nose, cheeks, chin)
3.5	Advanced	Wrinkles at rest, multiple, generalized, superficial; few, deep	Deep NL/ML folds, prominent jowels and SM, prominent neck strands	Violaceous E, numerous T, little uninvolved skin	Mostly rough little uninvolved skin	Many, large, generalized (nose, cheeks, chin, glabella)
4	Severe	Wrinkles throughout, numerous, extensively distributed, deep	Marked NL/ML folds, jowels and SM, neck redundancy, and strands	Deep, violaceous E, numerous T throughout	Rough throughout	Many, large, throughout (nose, cheeks, chin, glabella, forehead)

Abbreviations: E, erythema; EB, elastotic beads; ML, melolabial; NL, nasolabial; PO, periorbital; SM, submandibular; T, telangiectasi.

### 2.8 Statistical Analysis

The clinical trial analyzes utilized SPSS 21.0 (IBM, USA). The ACGS scores before and after treatment were expressed as mean ± standard deviation, and compared using a paired sample *t*‐test. A significance level of 0.05 with two tails was applied, with a statistically significant distinction considered as *p* < 0.05 at a 95% confidence level.

GraphPad Prism 8.0 (GraphPad Software, San Diego, CA) was also utilized for statistical analyses and graphing.

## 3 Results

### 3.1 Patterns of Action of RF on Ex Vivo Tissues: Higher Energy or Lower Frequency Results in a Faster Warming, Higher Endpoint Temperature, and Deeper Depth of Action

**FIGURE 1:**
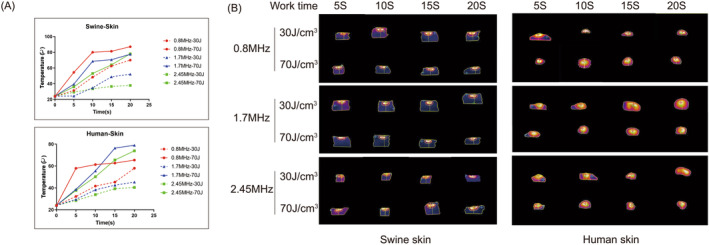
(A) The time–temperature curves show the use of each frequency at 30 and 70 J/cm^3^ for treating swine skin (top) and human skin (bottom). (B) Infrared thermograms displaying images of pig skin (left) and human skin (right) were captured at 5‐s intervals for a total of 20 s in each frequency. The energy levels used were 30 and 70 J/cm^3^.

The time–temperature curve (Figure 1A) reveals a common trend in both swine and human skin. As the frequency increases from 0.8 to 2.45 MHz, the rate of warming decreases and the endpoint temperature decreases accordingly. When using high energy for more than 10 s, all frequencies will heat the ex vivo tissue to over 50°C. However, when using 2.45 MHz, even with high energy, the temperature can be kept below 50°C if the usage time is less than 10 s. In human skin, 0.8 MHz exhibits faster heating with both high and low energy, even with low energy, it has a higher endpoint temperature and heats up sharply towards the end.

The infrared thermal imaging of swine skin (Figure 1B) and the heating depth results (Table 2) demonstrate that as the frequency increases, the puncture depth decreases. However, the differences between the three depths are minimal, all frequencies can reach a depth of 0.9 cm in ex vivo swine skin. All three frequencies can penetrate the human skin, but the differences are not statistically significant due to its thinness.

**TABLE 2:** The heating depth of swine skin and human skin was measured using a Thermal Imaging Camera at each frequency, using an energy of 30 and 70 J/cm^3^. The measurements were taken every 5 s during the treatment.

Duration of time	5 s	10 s	15 s	20 s	
The heating depth of swine skin (unit of measure: cm)					
0.8 MHz	30 J/cm^3^	0.58	0.84	0.92	1.1
	70 J/cm^3^	0.66	0.72	0.63	0.74
1.7 MHz	30 J/cm^3^	0.71	0.83	0.91	0.95
	70 J/cm^3^	0.78	0.91	0.92	0.96
2.45 MHz	30 J/cm^3^	0.8	0.78	0.74	0.82
	70 J/cm^3^	0.61	0.68	0.74	0.9
The heating depth of human skin (puncture depth/tissue thickness ratio)					
0.8 MHz	30 J/cm^3^	0.93	1	1	1
	70 J/cm^3^	1	1	1	1
1.7 MHz	30 J/cm^3^	0.73	1	1	1
	70 J/cm^3^	1	1	1	1
2.45 MHz	30 J/cm^3^	0.64	1	1	1
	70 J/cm^3^	1	1	1	1

**FIGURE 2:**
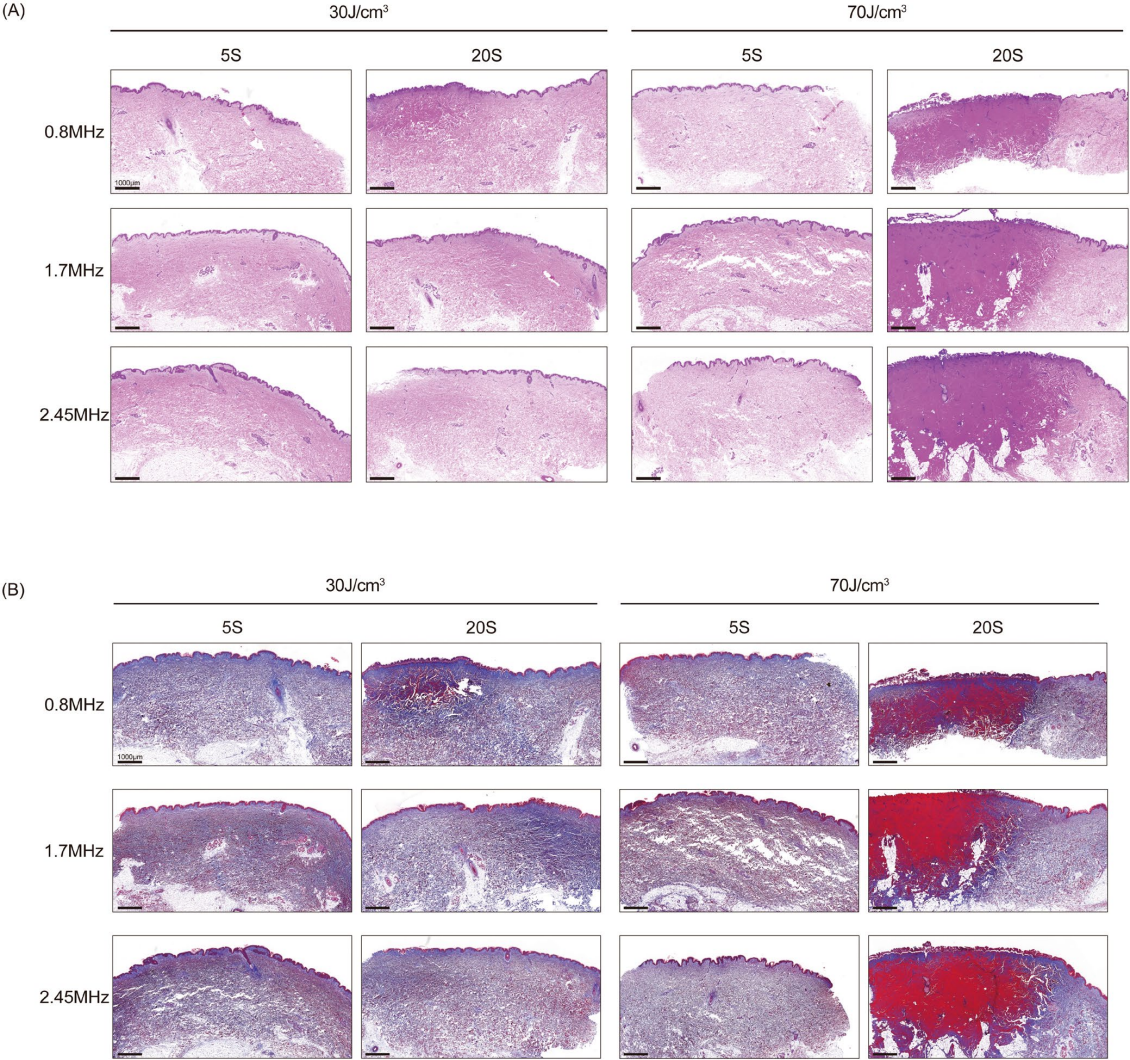
(A) The HE staining of human skin treated by each frequency at 30 and 70 J/cm^3^ at 5 s and 20 s. (B) The Masson staining of human skin treated by each frequency at 30 and 70 J/cm^3^ at 5 s and 20 s.

The HE staining demonstrates the thermal puncture depth and thermal effect resulting from RF treatment on human skin (Figure 2A). In human skin, when exposed to 70 J/cm^3^ of energy, all three frequencies are unsuitable for prolonged work, as they can lead to irreversible damage to the tissue layer. Additionally, at 0.8 MHz, even with at 30 J/cm^3^, can lead to prolonged work‐related thermal damage. However, in the short‐term work, using high energy in all three frequencies are relatively safe.

The changes in collagen fibers in the treated tissue are demonstrated by Masson staining (Figure 2B). For human skin, prolonged work at high energy in all frequencies can result in severe denaturation of collagen. 0.8 MHz has the greatest effect and may even cause thermal damage, followed by 1.7 and 2.45 MHz. Therefore, to ensure the desired effect while prioritizing safety, it is recommended to reduce working time for collagen remodeling. Furthermore, 2.45 MHz appears to be the safest option.

### 3.2 2.45 MHz Causes No Thermal Damage but is Still Effective In Vivo Animals

**FIGURE 3:**
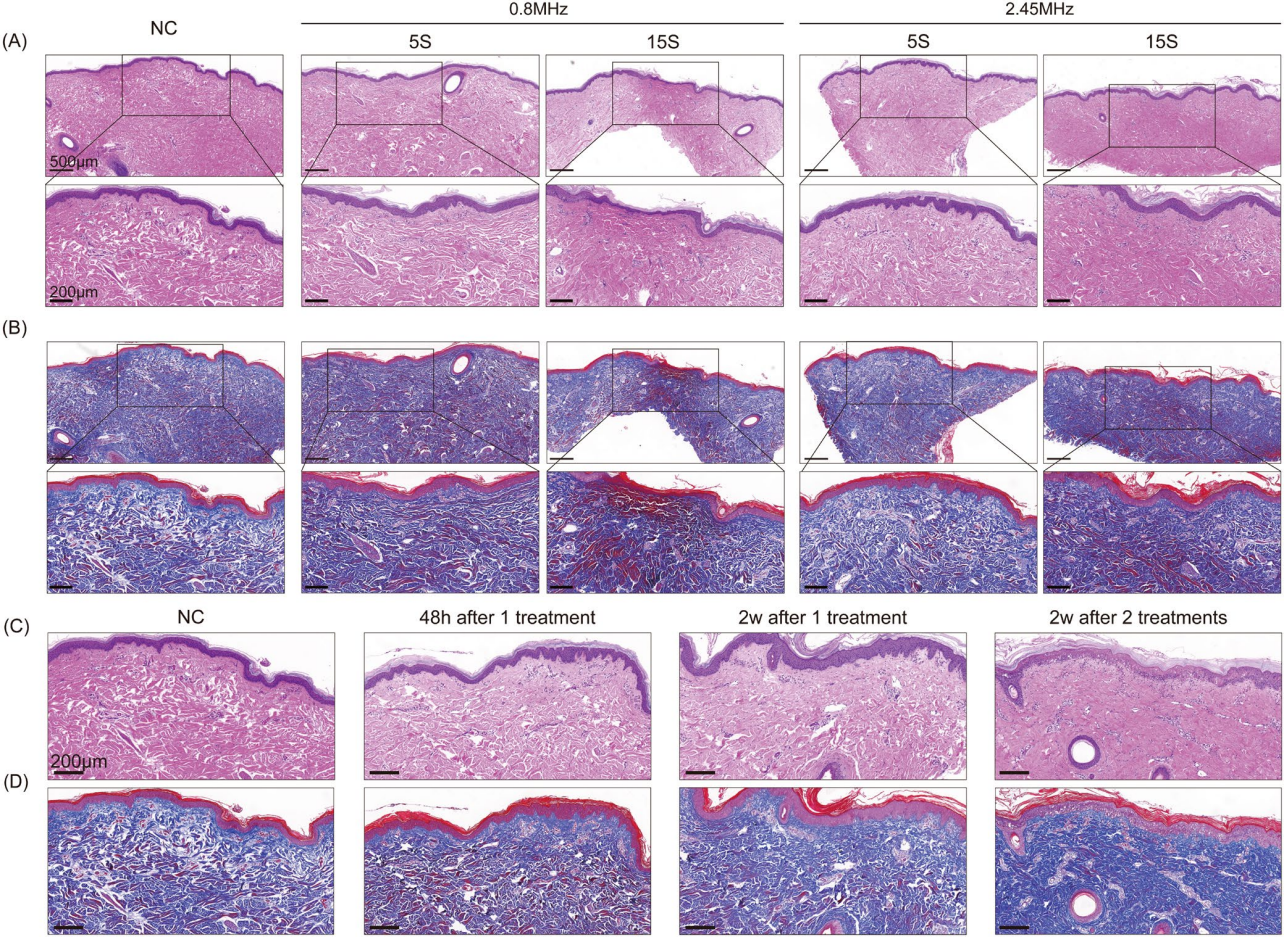
(A) The HE staining of dorsal skin of swine in vivo treated with 0.8 and 2.45 MHz at a duration of 5 s and 15 s, with an energy of 60 J/cm^3^. NC stands for the negative control group. (B) The Masson staining of dorsal skin of swine in vivo treated with 0.8 and 2.45 MHz at a duration of 5 s and 15 s, with an energy of 60 J/cm^3^. NC stands for the negative control group. (C) The HE staining of dorsal skin of in vivo swine treated with 2.45 MHz with an energy of 60 J/cm^3^ for 8 passes (from left to right, control group, 48 h after one treatment, 2 weeks after one treatment, 2 weeks after receiving two treatments at 2‐week intervals). (D) The Masson staining of dorsal skin of in vivo swine treated with 2.45 MHz with an energy of 60 J/cm^3^ for 8 passes (from left to right, control group, 48 h after one treatment, 2 weeks after one treatment, 2 weeks after receiving two treatments at 2‐week intervals.)

The histology results (Figure 3A,B) indicate that both 0.8 and 2.45 MHz penetrate the entire skin layer and cause collagen tightening. Nevertheless, 0.8 MHz poses a higher risk of irreversible damage. Even with the safest 2.45 MHz, a 15‐s duration of action still carries the risk of tissue damage.

Using 2.45 MHz with a power of 60 J/cm^3^ for 8 passes can effectively induce collagen tightening within 48 h, while also promoting collagen proliferation within 2 weeks (Figure 3C,D). These improvements become even more significant after undergoing two treatments. Additionally, this procedure is relatively safe and does not result in any irreversible damage.

**FIGURE 4:**
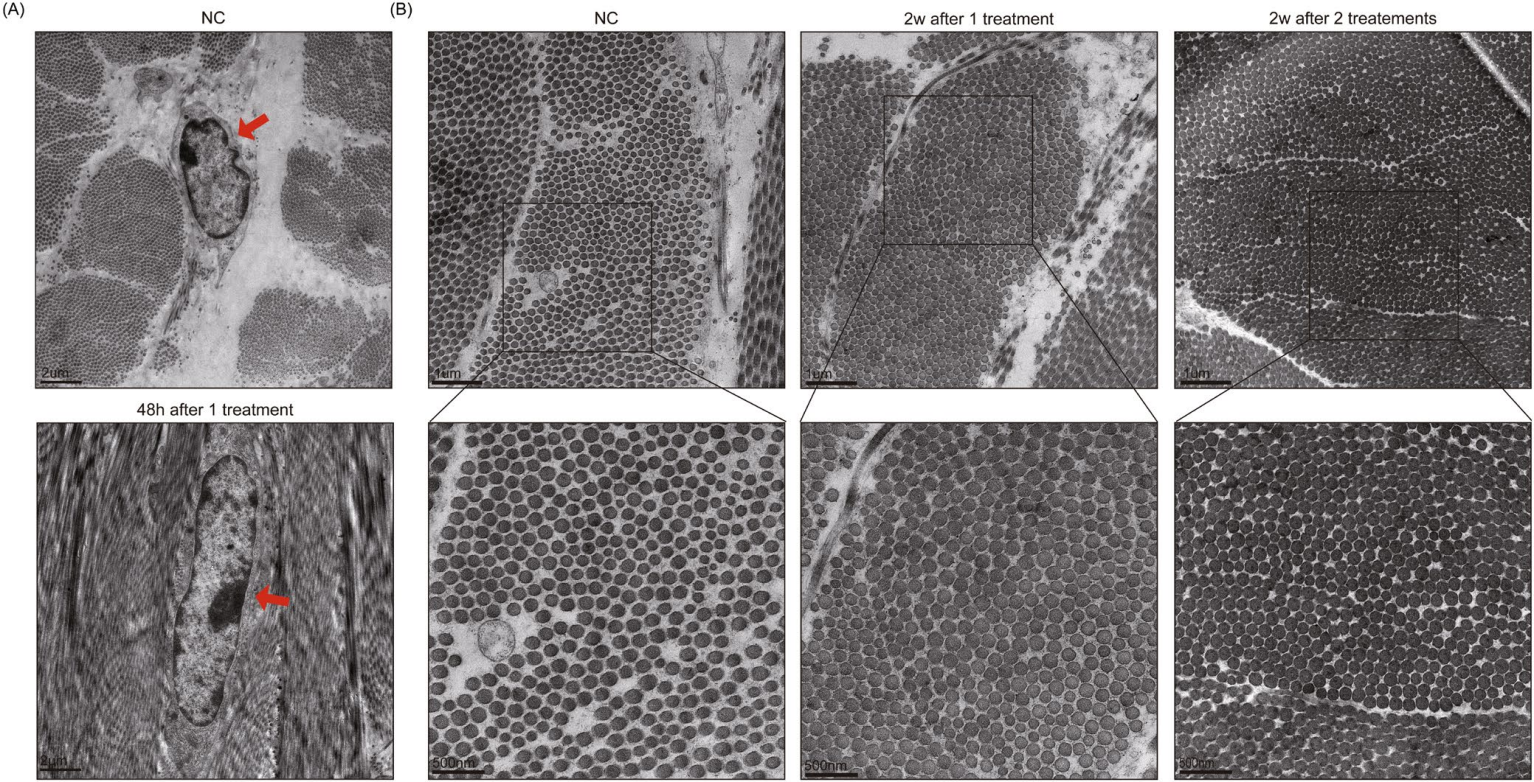
(A) The electron microscopy of fibroblast in dorsal skin of in vivo swine under two conditions: No treatment (NC) and 48 h after treated with 2.45 MHz, using an energy of 60 J/cm^3^ for 8 passes. The red arrows indicate the fibroblast. (B) The electron microscopy of collagen in dorsal skin of in vivo swine under three conditions: No treatment (NC), 2 weeks after treated with 2.45 MHz, using an energy of 60 J/cm^3^ for 8 passes, and 2 weeks after receiving two treatments at 2‐week intervals.

As can be seen in the results of electron microscopy, fibroblasts remain morphologically and structurally intact 48 h after treatment (Figure 4A). Two weeks later, there is a significant increase in collagen and this improvement is even more pronounced 2 weeks after receiving 2 treatments (Figure 4B).

### 3.3 Outstanding Rejuvenating Effect for Facial Fine Lines in Clinical Trials

In this clinical study, 46 patients were evaluated. Six of these patients did not complete the study: three were canceled due to inability to continue participation in the follow‐up study, two deferred treatment, and one was unable to have a follow‐up visit. Patient characteristics are presented in table (Table 3).

**TABLE 3:** Patient characteristics.

Number of subjects	46
Age, year (mean ± SD)	34.96 ± 8.96
Sex (male : female)	3 : 43
BMI (kg/m^2^) (mean ± SD)	21.28 ± 2.20
Fitzpatrick skin type	III–IV

**FIGURE 5:**
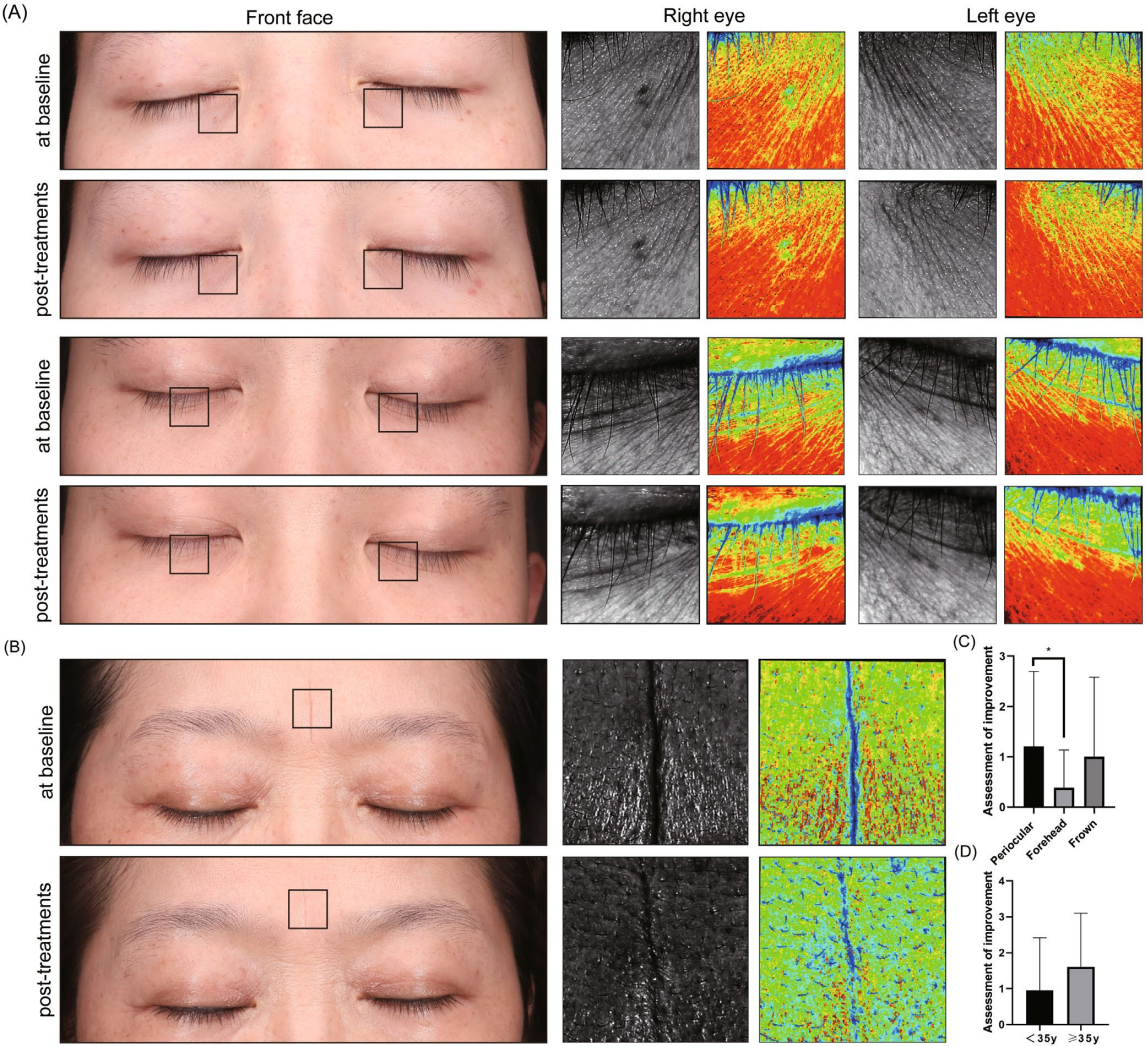
(A) Improvement of periocular wrinkles in the two patients at baseline and post‐treatments. (Top: 32‐year female pre and 3 months post three treatments; Bottom: 31‐year male pre and 3 months post three treatments) (B) Improvement of Frown lines in a patient at baseline and post‐treatments (56‐year female pre and 3 months post three treatments) (C) Independent observer assessment for baseline and after one treatment of improvement of wrinkles at various locations. (D) Improvement of periocular wrinkles after a single treatment in young versus middle‐aged patients.

Based on the comparison of the ACGS scale for 46 subjects before and after treatment, statistically significant differences were observed in wrinkles, laxity, texture, and pore size (*p* < 0.05). However, the changes are not noticeable in erythema (Table 4). During the follow‐up, the investigators found that this bipolar RF was very effective for the improvement of facial wrinkles, especially the fine lines around the eyes and at frown (Figure 5A,B).

**TABLE 4:** The ACGS scores at baseline and 3 months after the last treatment (post‐treatment).

Parameter	Wrinkles	Laxity	Erythema/telangiectasia	Texture	Pore
Baseline (mean)	1.73	1.62	1.10	1.37	1.30
Post‐treatment (mean)	1.15	1.12	1.10	1.01	1.07
Baseline‐post (mean ± SD)	0.57 ± 0.37	0.50 ± 0.33	0.01 ± 0.40	0.35 ± 0.30	0.16 ± 0.43
*p*‐value	< 0.0001	< 0.0001	0.85	< 0.0001	0.0177

In the GAIS, 18 participants showed “partially improved,” 16 showed “somewhat improved,” and 2 showed “very significantly improved.” The average GAIS assessment score is 3.33. The mean patient satisfaction score with the treatment outcome was 9.05 out of 10. Throughout the procedure, local pain was tolerable and no side effects were reported. The average pain score was 3.95 ± 2.39 out of 10.

Improvement of the treatment at different sites, as assessed by a blinded independent observer (Table 5), was most pronounced for periocular wrinkles and least for frontal wrinkles (Figure 5C). Analyses based on age groupings of patients concluded that the treatment was more effective for middle age. However, the difference is not significant (Figure 5D).

**TABLE 5:** An independent observer scored pre‐ and post‐treatment assessments of wrinkle improvement at various locations based on photographs.

Assessment of wrinkle improvement at different locations			
Location of wrinkles	Periocular	Frontal	Frown
Number of patients	39	26	9
After one treatment (%) (Mean ± SD)	12.05 ± 14.90	3.85 ± 7.52	12.22 ± 16.41
After three treatment (%) (Mean ± SD)	11.32 ± 21.33	2.80 ± 6.14	2.22 ± 4.41

Furthermore, in the patient's video data, we observed that the treatment also had a positive impact on the patient's dynamic wrinkles (see S1).

## 4 Discussion

Skin aging is a natural process influenced by genetic factors and various environmental factors, including natural aging and external influences. It is mainly characterized by thinning of the skin, reduction in the number of fibroblasts, and collagen fibers in the dermis, deformation of elastic fibers, etc. [11]. The main clinical signs include skin laxity, dullness, roughness, enlarged pores, and wrinkles. Traditional treatments like myofascial lift facelift, buried threads, and fillers have been effective, but the risks of surgery, complications, side effects, and long recovery times have deterred many patients from choosing these treatments, especially patients with mild facial laxity and fine lines. On the other hand, RF is widely used in facial rejuvenation treatments because of its ease of use, efficiency, less invasive, and safety.

RF for skin rejuvenation affects tissues primarily through thermal effects. RF treatment targets the deeper layers by causing selective thermal effect to the tissues due to their resistive nature. However, whether it is thermal damage or just stimulation has always been controversial. By far the most widely accepted principle of RF is that it mainly acts on the collagen at the loose connective tissue, causing denaturation of the collagen's triple helix structure through heat, resulting in tissue contraction. Additionally, the heat‐induced denaturation of collagen triggers a trauma‐healing response in the dermis, resulting in the formation of new and more interlocking collagen fibers. This stimulation promotes continuous collagen renewal and remodeling [12, 13]. A temperature of 85°C for 1 ms or 67°C for 3 s is enough to change the structure of collagen protein. However, according to the research results of Kreindel and Mulholland, a temperature of 50°C–80°C will cause coagulative necrosis of soft tissue and collagen protein shrinkage. A temperature of 45°C–50°C will cause protein (including collagen) to undergo conformational changes and induce cell death. A temperature of 37°C–44°C can promote metabolism and accelerate other natural processes [8]. Additionally, when temperature is raised to 43°C for 10 min, it promotes the proliferation of human dermal fibroblasts [7]. Therefore, many authors have suggested that, in order to avoid damage to the epidermis and soft tissues, the temperature should be properly controlled, preferably below 43°C [14]. This study confirmed that thermal stimulation, rather than thermal damage, is sufficient to achieve rejuvenation effects through various experiments. It was concluded from the time–temperature curve that using 2.45 MHz at high energy will not exceed 50°C within 10 s of exposure. In subsequent histological experiments, it was also found that no thermal damage occurs within 5 s of exposure at high energy, and the risk of thermal damage was only observed after 15 s. Furthermore, in in vivo experiments, thermal stimulation is enough to promote collagen proliferation.

The clinical effects of bipolar RF have been previously proven [15, 16]. However, there is considerable diversity in the parameters that are applied. Without detailed guidance on parameters, many adverse effects can occur. Currently, the frequency of bipolar RF available on the market is mainly below 3 MHz, with the majority being 1 MHz or under 1 MHz [17, 18]. However, our study investigates multiple frequencies ranging from 0.8 to 2.45 MHz through ex vivo and in vivo experiments. The aim is to determine the most effective and safe frequency, as well as the optimal energy and duration of action for facial fine lines treatment. In the ex vivo study, different frequencies in the three frequencies resulted in a slight difference in the depth of action. However, this difference was not significant in the in vivo study, possibly due to the diffusion of heat with the involvement of blood circulation. By ex vivo and in vivo study, we observed instability in 0.8 MHz, which may be attributed to its low frequency. Of all the frequencies, 2.45 MHz proved to be the most stable. Therefore, we recommend using 2.45 MHz for the treatment of fine lines and the action duration of no more than 15 s. In in vivo studies, collagen remodeling and proliferation were evident after treatment, which coincided with clinical results.

Unlike previous studies, we found that bipolar RF at 2.45 MHz was effective in treating mild facial laxity, such as fine lines, particularly periocular lines, but not as effective in cases of moderate to severe facial laxity. The results suggest that treatment may be more effective in patients over the age of 35, possibly because fine lines on the face are more pronounced in middle‐aged individuals. Surprisingly, we also discovered that this treatment is effective for dynamic wrinkles. Furthermore, it is worth noting that the majority of clinical research on bipolar RF has primarily focused on European and American populations. Unfortunately, there is a shortage of thorough findings from clinical trials regarding facial treatments for Asian populations. We hope that our research can help fill this gap.

## 5 Limitations

While research has extensively examined the thermal effects of RF, its non‐thermal effects, such as electrical stimulation, remain largely unexplored. It should be emphasized that there are many more reasons that need to be researched. Our clinical trials have shown that RF effectively improves dynamic wrinkles, indicating a potential impact on facial muscles. It has been confirmed that RF treatment stimulates changes in cytokines and growth factors [19], including heat shock proteins (HSPs) [20]. This research primarily concentrated on confirming the histological impacts of bipolar RF, without delving into its molecular effects. The mechanism of RF anti‐aging has not been thoroughly studied, and future follow‐up studies are needed.

Due to the varying impedance of individuals' skin tissue, it is currently not possible to determine an exact parameter for treating facial fine lines. After conducting our research, we can only offer a range of treatment parameters that are both safe and effective, we still need to personalize the treatment parameters by closely observing the patient during the treatment.

Additionally, this clinical study was a single‐center, single‐arm, controlled experiment and the number of subjects in clinical trials was small. Therefore, in the future, more clinical trials with larger sample sizes need to be conducted in order to further confirm the efficacy.

## 6 Conclusion

This study confirmed that thermal stimulation, rather than thermal damage, is sufficient to achieve rejuvenation effects. The bipolar RF device has been found to effectively and safely rejuvenate facial fine lines, thanks to its ability to penetrating the full skin layer and promote collagen remodeling, as evidenced by this study. We recommend using a frequency of 2.45 MHz, energy levels of 50–80 J/cm^3^, and an action duration about 8 s for treating fine skin lines.

## Author Contributions

**Min Yao** and **Wei Ni:** conceptualization. **Wei Ni** and **Yubing Bai:** methodology. **Yubing Bai** and **Wei Ni:** validation. **Yubing Bai** and **Yiqiu Zhang:** formal analysis. **Yubing Bai**, **Zhixuan Jiang**, and **Shengzhe Zhou:** investigation. **Yubing Bai** and **Yiqiu Zhang:** data curation. **Yubing Bai** and **Yiqiu Zhang:** data analysis. **Yubing Bai:** writing – original, draft preparation. **Yubing Bai**, **Wei Ni**, and **Min Yao:** writing – review and editing. **Yubing Bai:** visualization. **Min Yao** and **Wei Ni:** project administration. All authors have read and agreed to the published version of the manuscript.

## Ethics Statement

The ex vivo and in vivo experiments were approved and supervised by the Institutional Ethics Committee of Shanghai Jiao Tong University School of Medicine Affiliated Ninth People's Hospital (code no. SH9H‐2023‐A835‐1 for swine tissue and code no.SH9H‐2020‐T138‐2 for human tissue). The clinical trial is approved by the Ethic Board of Shanghai Jiao Tong University School of Medicine Affiliated Ninth People's Hospital, Shanghai, China (SH9H‐2023‐T155‐1) and registered at the Chinese Clinical Trial Registry (ChiCTR2300071155). All subjects have signed the institutional informed consent form, which includes consent for social media publication.

## Conflicts of Interest

The authors declare no conflicts of interest.

## Supporting information


**Video S1.** Improvement of dynamic frown lines in a patient at baseline and post‐treatment (56‐year female pre and 3 weeks after 1 treatments) and the improvement of dynamic frontal lines in a patient at baseline and post‐treatment (45‐year female pre and 3 weeks after 1 treatments).

## Data Availability

The data that support the findings of this study are available on request from the corresponding author upon reasonable request.
